# A Multicomponent Intervention Program With Overweight and Obese Adolescents Improves Body Composition and Cardiorespiratory Fitness, but Not Insulin Biomarkers

**DOI:** 10.3389/fspor.2021.621055

**Published:** 2021-02-22

**Authors:** Letícia de Borba Schneiders, Caroline Brand, Leticia Borfe, Anelise Reis Gaya, Javier Brazo-Sayavera, Jane Dagmar Pollo Renner, Cézane Priscila Reuter

**Affiliations:** ^1^Postgraduate Program Master and Doctorate in Health Promotion, University of Santa Cruz do Sul, Santa Cruz do Sul, Brazil; ^2^Postgraduate Program Master and Doctorate in Human Movement Sciences, Federal University of Rio Grande do Sul, Porto Alegre, Brazil; ^3^Department of Sports and Computer Sciences, Universidad Pablo de Olavide, Seville, Spain

**Keywords:** adolescents, obesity, cardiorespiratory fitness, exercise, biomarkers

## Abstract

**Objective:** To verify the effect of a multicomponent intervention with overweight/obese adolescents on physical fitness, body composition, and insulin biomarkers.

**Methods:** A quasi-experimental study with 37 adolescents, aged 10 to 17 years, of both sexes, overweight and obese, allocated in two groups (Intervention—IG Group, *n* = 17; Control—GC Group, *n* = 20). The IGs were submitted to a multicomponent intervention for 6 months (three weekly sessions) consisting of physical exercises (sports, functional circuit, recreational, and water activities) and nutritional and psychological guidance. Participants were assessed before and after intervention on body composition [body mass index (BMI), body fat, waist circumference, and waist-to-hip ratio (WHR)], physical fitness [cardiorespiratory fitness (CRF) and abdominal strength], and biomarkers of insulin (glucose, insulin, evaluation of the homeostasis model of insulin, and resistin resistance). The prevalence of responders in both groups was obtained according to the theoretical model applied in previous studies similar to this one to determine the cutoff points for response to intervention. Poisson regression was used to verify the difference in the prevalence ratio (PR) of the interviewees between the groups.

**Results:** The responders' prevalence between groups CG and IG showed significant differences for body fat (CG = 30.0%; IG = 70.6%; PR = 1.396; *p* < 0.001), WHR (CG = 30.0%; IG = 76.5%; PR = 1.730; *p* < 0.001), and CRF (CG = 15.0%; IG = 52.5%; PR = 1.580; *p* < 0.001).

**Conclusions:** A 6-month multicomponent intervention program improved certain body composition parameters and the CRF of overweight and obese adolescents but did not improve insulin biomarkers.

**Clinical Trial Registration:** Clinical Trials under Protocol ID: 54985316.0.0000.5343.

## Introduction

Nowadays, excess weight contributes to the increase in factors that lead to morbidity and mortality, such as sleep disorders, eating disorders, physical inactivity, and sedentary behaviors (Jääskeläinen et al., 2014). It is during adolescence when behaviors that will last into adult life are developed, contributing inadequately to the development of future metabolic and cardiovascular diseases such as type 2 diabetes mellitus, dyslipidemia, and hypertension. Finding alternatives to prevent this condition is extremely important, for young people with obesity as for those with overweight (Lakshman et al., 2015).

The increase in body fat accelerates the process of chronic inflammation of adipose tissue, causing hormonal dysfunction and leading to a high release of inflammatory cytokines (Galic et al., 2010). Several cytokines expressed in obesity can manipulate the action of the insulin hormone (Wensveen et al., 2015), such as resistin, which inhibits the signaling of its receptors (Yamasaki et al., 2018) and increases lipolysis in cells of fat, causing a greater release of fatty acids (Nakamura et al., 2014). As a consequence, high levels of free fatty acids can cause insulin resistance in tissues, such as liver and muscles (Zhang et al., 2011), a worrying fact considering that insulin resistance is known as the first phase for the development of cardiometabolic diseases (Patel et al., 2013).

There is evidence that physical exercise can reduce the abovementioned inflammation (Brunelli et al., 2015; Lopes et al., 2016), as it is characterized as a viable alternative to reduce body fat and improve cardiorespiratory fitness (CRF), being able to induce physiological, endocrine, and cardiovascular adaptations (Cordero et al., 2014; Medrano et al., 2017). Thus, physical exercise exerts positive changes and has become a protagonist in the processes of preventing excess weight and treating obesity, modifying the metabolic hormones related to chronic inflammation (Ping et al., 2018). Results of physical exercise in health parameters are mainly evidenced on mean values and considering groups, but there is an individual variability in their response that has not yet been explored sufficiently in children and adolescents (Bouchard et al., 2012; Alvarez et al., 2017; Álvarez et al., 2017). This means that although the same stimulus is being applied, some individuals may respond in different ways, obtaining positive or negative responses after an intervention with physical exercise, characterizing them as responders and non-responders (Montero and Lundby, 2017). In view of the need to develop strategies that can match the complexity presented in the diagnosis of excess weight, interventions that include multicomponent actions, encompassing social, environmental, curricular, and educational aspects, deserve to be highlighted and are strongly recommended (Rajmil et al., 2017).

In this sense, multicomponent intervention programs are considered one of the best options for the treatment and reduction of excess weight, as they work with actions from different professional areas, with the purpose of promoting behavioral adaptations related to the levels of physical activity and healthy eating habits (Hampl et al., 2016). Bearing in mind that a multicomponent intervention program with sessions of aerobic and anaerobic exercise and nutritional and psychological guidance can promote improvements in the health parameters of individuals with overweight and obesity, the present study aims to verify the effect of a multicomponent intervention with overweight/obese adolescents in physical fitness, body composition, and insulin biomarkers.

## Materials and Methods

### Study Design

This is a quasi-experimental study, part of the project “Obesity in elementary school students: a multicomponent intervention study—Phase III” developed with overweight/obesity adolescents from public and private schools, of both sexes, aged between 10 and 17 years old. The multicomponent intervention program consisted of physical exercise sessions and psychological and nutritional guidance. The project was developed in 2016, over 6 months. All procedures were approved by the Scientific Council of the Research Unit that leads the project under number CAAE: 54985316.0.0000.5343 and opinion number: 1.498.338, and are registered at Clinical Trials under Protocol ID: 54985316.0.0000.5343. All participants presented the free and informed consent form signed by the parents and guardians, along with the consent form signed by the adolescents' themselves.

### Participants and Procedures

Participants for the intervention group (IG) were invited among the overweight/obese adolescents included in the cross-sectional survey “Schoolchildren's health—phase IV” with overweight and obesity. To increase the sample size in the IG, we invited individuals from the community with the same characteristics, through disclosures and communication strategies (radio, newspapers, and social networks); interested ones should contact the project coordinators. Visits and meetings were held in schools in the municipality, with a pedagogical team, parents, and guardians, to present the intervention program. After that, schools indicated which students would take part in the study.

To join the control group (CG), schoolchildren with similar characteristics were invited, such as being overweight or obese and belonging to schools in the municipality. Participants were selected by convenience criteria, according to the available data from the research. There was no intervention in the lifestyle of this group.

**Figure 1:**
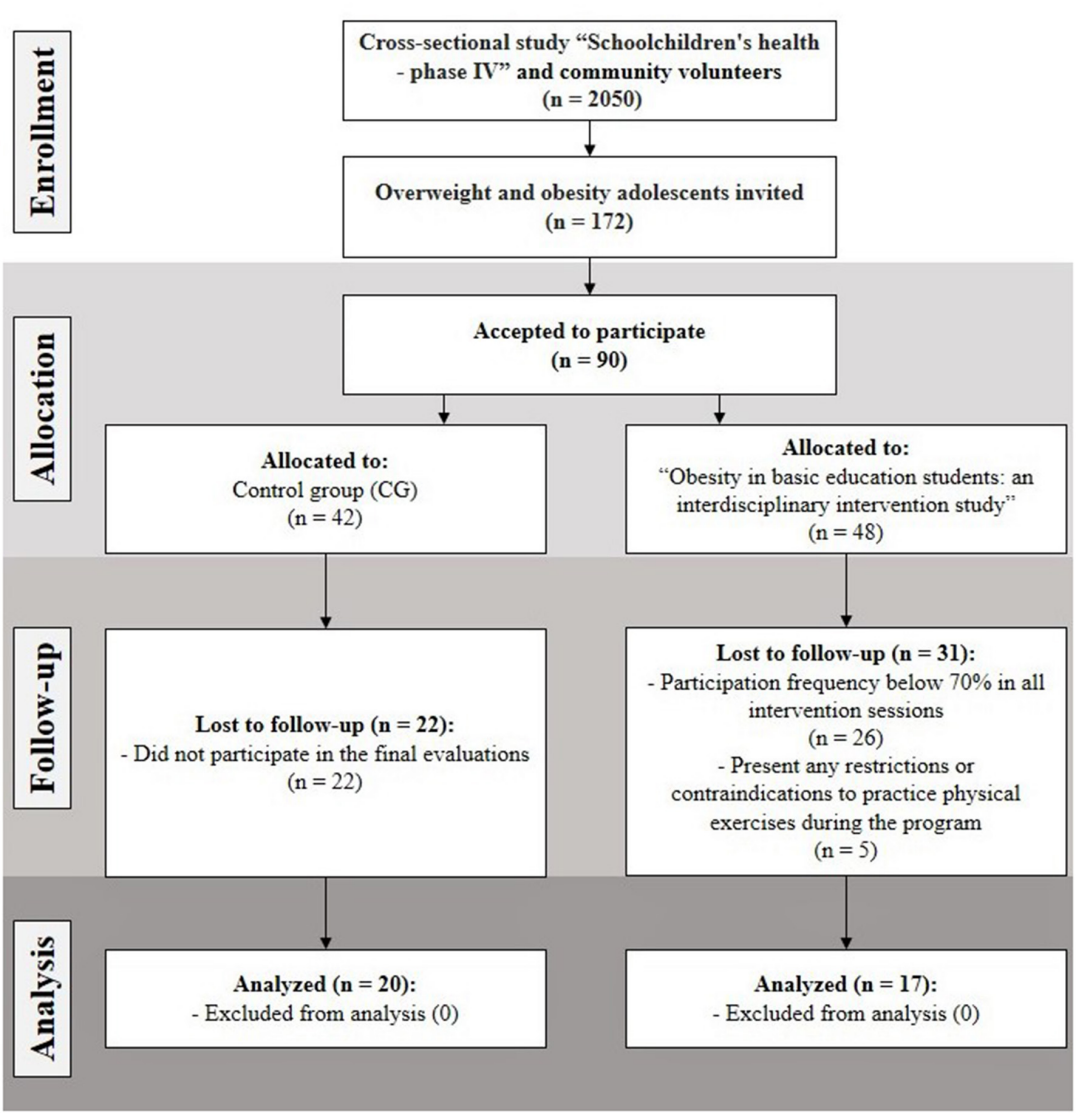
Sample flowchart.

The sample size calculation was performed in G ^*^ Power program (Faul et al., 2007). Based on the estimates by Hulley (2013), it was considered a test power of 0.8, an effect of 0.30, and a significance level of 95%, requiring at least 11 subjects for the CG and 11 subjects for the IG. Figure 1 shows the selection of control and intervention groups at baseline, considering the inclusion and exclusion criteria.

### Multicomponent Intervention Program

The IG took part in a multicomponent program with activities distributed in 2-h sessions, three times a week (Mondays, Wednesdays, and Fridays) in the afternoon, after the school schedule. The intervention was carried out over 6 months and included the participation of a multicomponent team with professionals of Physical Education, Pharmacy, Physiotherapy, Nutrition, Psychology, Nursing, and Medicine, among others. Physical exercise sessions and psychological and nutritional guidelines were applied.

### Physical Exercise Intervention

The following physical exercise plans were developed and applied: Monday sessions consisted of a warm-up, stretching, walking, and sports activities (futsal, handball, basketball, and jiu-jitsu). On Wednesdays, sessions consisted of water activities such as water aerobics, recreational activities, and swimming for beginners. On Fridays, aerobic workouts, a circuit of functional and resistance workouts, as well as breathing exercises were proposed. During the exercise, heart rate (HR) was controlled using HR monitors (Polar Cardiac Monitor—FT1), and training intensity was defined to induce HR between 50 and 70% of maximum HR, considering the Karvonen equation (maximum HR = 220 – age). More details and information on physical activity and sports sessions can be accessed in the Supplementary Document.

### Nutritional Education Intervention

The nutritional sessions were held once a week, more precisely on Wednesdays, consisting of food re-education with activities that aimed to know foods; reduce fats, sugar, and sodium consumption; choose the healthiest foods; and identify the daily portions; among others. Conversations, games, videos, teaching materials, and practical classes were held with healthy recipes for adolescents.

### Psychological Intervention

Psychological intervention sessions were held once a week, on Mondays, with collective guidance and cognitive training, using techniques focused on the thoughts and feelings aroused by obesity. Relaxation methods were also developed to restructure these thoughts (Lüdtke et al., 2018).

## Measurements

### Maturational Stages

As an indicator of sexual maturation, Tanner's adapted staging method (Tanner, 1962) was applied and a self-assessment test was performed individually, using images that represent the stage of development of pubic hair. Pubic hair is evaluated for its characteristics, quantity, and distribution, in both sexes. The stages of development are classified from 1 to 5, with stage 1 being the infantile phase (pre-pubertal) and stage 5 being the adult phase (post-pubertal). Thus, stages 2, 3, and 4 characterize the pubertal period.

### Anthropometric Measures and Body Composition

To verify weight and height, a calibrated anthropometric scale was used, composed of a coupled stadiometer. Body mass index (BMI) was calculated through the equation BMI = weight/height^2^ (kg/m^2^), to later classify the results through the percentile curves of WHO (1988), which considers overweight individuals with *p* ≥ 85 and *p* < 97 and with obesity *p* ≥ 97. A measuring tape with a resolution of 1 mm not extensible was used to measure waist circumference (WC) and hip circumference (HC). Then, waist-to-hip ratio (WHR) was calculated following the equation: waist (cm)/hip (cm). A skinfold caliper (Lange, Beta Technology Inc., Houston, USA) was used to assess triceps and subscapular skinfolds and calculate the body fatness following the equation proposed by Slaughter et al. (2016).

### Aerobic and Muscular Fitness

The assessments of aerobic and muscular fitness were performed according to the protocols and cutoff points defined in the Project Sport Brazil manual (Gaya and Gaya, 2016), including the CRF test and abdominal strength (sit up). The CRF test consists of a 6-min running/walking test, in which participants must complete the largest number of laps, running or walking, on an official athletics track, marked with signage indicated in meters. The CRF was evaluated by the number of laps performed and the distance covered until the final test time for those who did not complete a lap. A complete lap on the track consists of 400 m covered, so it was calculated for the test classification: the number of laps covered × 400 m + the remaining distance covered in meters if the subject did not complete the lap in the last minute of the race. The abdominal strength test was performed with the subjects lying on a mat, in supine position, with knees flexed, arms crossed over the chest, and ankles fixed on the floor by the evaluator. The movement consisted of flexing the trunk until it touched thighs with elbows, returning to the initial position, accounting for the largest number of repetitions of the complete movement in 1 min. From this, the cutoff points stratified by sex and age were used, established in two categories for both tests: (1) health risk zone when below expectations and (2) healthy zone when equal or above expectations.

### Biochemical Assays

A trained professional extracted the blood for biochemical analyses at the Laboratory of Exercise Biochemistry, at the University of Santa Cruz. Ten milliliters of blood was collected in the brachial vein, of which 5 ml was used in the vacutainer tube without anticoagulant (for analysis of biochemical, inflammatory, and metabolic indicators) and 5 ml of whole blood for EDTA vacutainer tube (for blood count measurement). Participants were instructed to remain fasting for 12 h. Insulin was measured through the serum (Architect i2000SR, Abbott Laboratories, Chicago, USA). The homeostasis model assessment of insulin resistance (HOMA-IR) was calculated using the formula HOMA = fasting glucose (mmol/L) × insulin (μU/L)/22.5 (Huang et al., 2002); the results were classified according to the cutoff established by Rocco et al. (2011) considering >1.65 for girls and >1.95 for boys. The measurement of serum resistin levels was performed using a serum sample (Luminex® Platform, Life Technologies, Inc., São Paulo, BRA) and an outsourced Laboratory performed it.

### Statistical Analysis

Shapiro–Wilk tests were conducted to test the normality and Levene's test was used for homogeneity of variance. Statistical analyses (Student's *t*-test and chi-square) were performed to rule out possible differences from the total sample. For this, the two groups were matched for sex, age, and anthropometric variables (WC, BMI, and body fat). The *t*-test for independent samples and the chi-square test was used to compare groups' characteristics. The effect size between pre- and post-intervention was also calculated using the means and standard deviation of the groups through Cohen's *d*, with results classified as >0.2, small effect; >0.5, moderate effect; >0.8, large effect; and >1.2, very large effect (Cohen, 1992).

The prevalence of responders in the outcome variables in both groups was obtained according to the theoretical model applied in previous studies with results of anthropometric, physical, and biochemical variables, considering the Δ% effect. The Δ% values obtained in previous interventions were considered acceptable, apparently with the same procedures and variables to determine the parameters and cutoff points for intervention responders (*R*). For this study, it was considered the follow cutoff point to determine responders and non-responders in each one outcome: BMI (change in cutoff points *R* > −3.29%) and WC (change in cutoff points *R* > −3.97%) were based on Ranucci et al. (2017), WHR (change in cutoff points *R* > −3.57%) was based on Nourse et al. (2015), and body fatness (change in cutoff points *R* > −10.42%) was based on Nardo Junior et al. (2016). For abdominal strength (change in cutoff points *R* > 12.2%), it was based on Silva et al. (2013) while Oliveira et al. (2017) considered a reference for CRF (change in cutoff points *R* > 10%). For glucose (change in cutoff points *R* > −5.88%), insulin (change in cutoff points *R* > −34.88%) and HOMA-IR (change in cutoff points *R* > −9.95%) were based on Garanty-Bogacka et al. (2011), and resistin (change in cutoff points *R* > −16%) was based on McFarlin et al. (2013). Poisson regression was used to verify the difference in the prevalence of responders between IG and CG. All analyses were performed on IBM SPSS 23.0 (SPSS, Inc., Chicago, Illinois, USA). The level of statistical significance was considered as *p* < 0.05.

## Results

Participants' characteristics are shown in Table 1. The sample consisted of 43.2% boys and 56.8% girls. There was a significant difference in WHR (*p* = 0.02) and in the sexual maturation stage (*p* = 0.05) for adolescents at baseline.

**Table 1:** Participants' descriptive characteristics by group.

	**Baseline**			**Post-intervention**		
	**CG** ** (*n* = 20)**	**IG** ** (*n* = 17)**	***p***	**CG** ** (*n* = 20)**	**IG** ** (*n* = 17)**	***P***
Age (years)	13.15 (1.66)	12.94 (1.14)	0.67	13.55 (1.73)	13.29 (1.21)	0.61
Weight (kg)	67.72 (15.25)	72.49 (14.47)	0.34	68.05 (15.99)	71.19 (14.45)	0.54
Height (m)	1.56 (0.09)	1.60 (0.10)	0.29	1.59 (0.09)	1.63 (0.09)	0.26
BMI (kg/m^2^)	27.44 (4.16)	28.26 (4.28)	0.56	26.63 (4.90)	26.75 (4.26)	0.94
Body fat (%)	29.22 (6.00)	32.26 (6.23)	0.14	29.32 (7.62)	27.25 (7.92)	0.43
WC (cm)	81.12 (8.52)	87.30 (10.58)	0.06	79.77 (10.29)	79.74 (8.30)	0.99
HC (cm)	100.67 (10.16)	100.93 (7.04)	0.93	97.76 (11.50)	101.87 (10.18)	0.26
WHR (cm)	0.80 (0.07)	0.86 (0.07)	0.02	0.82 (0.09)	0.78 (0.04)	0.11
CRF (m)	809.25 (147.06)	810.00 (140.85)	0.99	797.35 (172.26)	889.29 (197.59)	0.14
Abdominal strength (rep)	21.10 (6.75)	19.29 (12.88)	0.53	22.10 (7.75)	22.47 (8.39)	0.89
Glucose (mmol/L)	4.98 (0.33)	4.90 (0.32)	0.44	4.93 (0.35)	4.83 (0.41)	0.42
Insulin (μU/L)	6.63 (3.19)	7.56 (3.60)	0.41	5.83 (3.84)	8.05 (6.43)	0.20
HOMA-IR	1.47 (0.71)	1.4 (0.76)	0.48	1.29 (0.87)	1.71 (1.31)	0.25
Resistin (ng/ml)	32.79 (14.05)	28.03 (9.89)	0.25	22.08 (8.58)	27.15 (14.97)	0.21
	***n*** **(%)**					
Maturational stages^*^						
Not matured	7 (35.0)	1 (5.9)	0.05	3 (15.0)	1 (5.9)	0.30
Continuing maturation	9 (45.0)	14 (82.4)		14 (70.0)	10 (58.8)	
Matured	4 (20.0)	2 (11.7)		3 (15.0)	6 (35.3)	
HOMA-IR^*^						
Normal	13 (65.0)	10 (58.8)	0.48	15 (75.0)	10 (58.8)	0.24
Not normal	7 (35.0)	7 (41.2)		5 (25.0)	7 (41.2)	

*Data expressed as mean and standard deviation; t-test for independent samples; significant differences for p <0.05; CG: control group; IG, intervention group; BMI, body mass index; WC, waist circumference; HC, hip circumference; WHR, waist-to-hip ratio; CRF, cardiorespiratory fitness; HOMA-IR, homeostasis model assessment of insulin resistance*.

* *Data expressed as frequencies and percentages; chi-square test; significant differences for p < 0.05*.

Table 2 shows the effect of time, group, and interaction on the studied variables. Significant interaction (time × group) was found in body fat (*p* = 0.004), WHR (*p* = 0.003), CRF (*p* = 0.029), and resistin (*p* = 0.008). When analyzing the effect sizes of the intervention using Cohen's *d*, it is observed that the changes in the IG had a very large effect size on WHR (*d* = 1.33), a large effect size on body fat (*d* = 1.01) and WC (*d* = −0.86), and a moderate effect size on CRF (*d* = 0.65). On the other hand, resistin showed a large effect size (*d* = 1.09) and HC presented a moderate effect size (*d* = −0.50) for the changes observed in the CG.

**Table 2:** Effect of intervention on time, group, and interaction on indicators of body composition, aerobic and muscular physical fitness, insulin resistance, and serum resistin levels.

				Mixed ANOVA^*^								
				**Time**			**Group**			**Time** **×** **Group**		
		**Δ%**	***d***	***F***	***p***	**Eta**	***F***	***p***	**Eta**	***F***	***p***	**Eta**
BMI (kg/m^2^)	CG	−3.10	−0.60	4.543	0.041	0.124	0.038	0.847	0.001	0.029	0.865	0.001
	IG	−4.86	−0.48									
Body fat (%)	CG	−0.16	0.02	6.825	0.014	0.176	0.089	0.767	0.003	9.823	0.004	0.235
	IG	−16.09	−1.01^**^									
WC (cm)	CG	−1.53	−0.18	2.531	0.121	0.073	0.613	0.439	0.019	3.497	0.071	0.099
	IG	−8.08	−0.86^**^									
HC (cm)	CG	−2.88	−0.50*	0.830	0.369	0.025	0.199	0.659	0.006	3.000	0.093	0.086
	IG	1.03	0.11									
WHR (cm)	CG	1.98	0.11	1.175	0.286	0.035	0.382	0.541	0.012	10.362	0.003	0.245
	IG	−8.90	1.33^***^									
CRF (m)	CG	−0.95	−0.09	1.939	0.173	0.057	1.754	0.195	0.052	5.230	0.029	0.140
	IG	9.74	0.65^*^									
Abdominal strength (rep)	CG	5.19	0.13	0.341	0.563	0.011	0.032	0.859	0.001	0.078	0.781	0.002
	IG	50.36	0.33									
Glucose (mmol/L)	CG	−0.84	−0.16	5.522	0.025	0.147	1.356	0.253	0.041	0.196	0.661	0.006
	IG	−1.14	−0.16									
Insulin (μU/L)	CG	−7.68	−0.24	0.655	0.424	0.020	0.633	0.549	0.011	0.134	0.716	0.004
	IG	3.99	0.10									
HOMA-IR	CG	−8.04	−0.23	0.227	0.637	0.007	0.194	0.662	0.006	0.001	0.972	0.000
	IG	3.80	0.11									
Resistin (ng/ml)	CG	−29.40	1.15^**^	0.244	0.625	0.008	0.145	0.706	0.005	7.982	0.008	0.198
	IG	−5.15	−0.10									

BMI, body mass index; WC, waist circumference; HC, hip circumference; WHR, waist-to-height ratio; CRF, cardiorespiratory fitness; HOMA-IR, homoeostasis model assessment of insulin resistance. Δ%: percentage delta; Cohen's d;

* moderate d effect size (d > 0.5);

** large d effect size (d > 0.8);

*** *very large d effect size (d > 1.2); F: Mixed ANOVA; p: significance level <0.05; Eta, eta squared effect; CG, control group; IG, intervention group; adjusted for sex, age and sexual maturation*.

**Figure 2:**
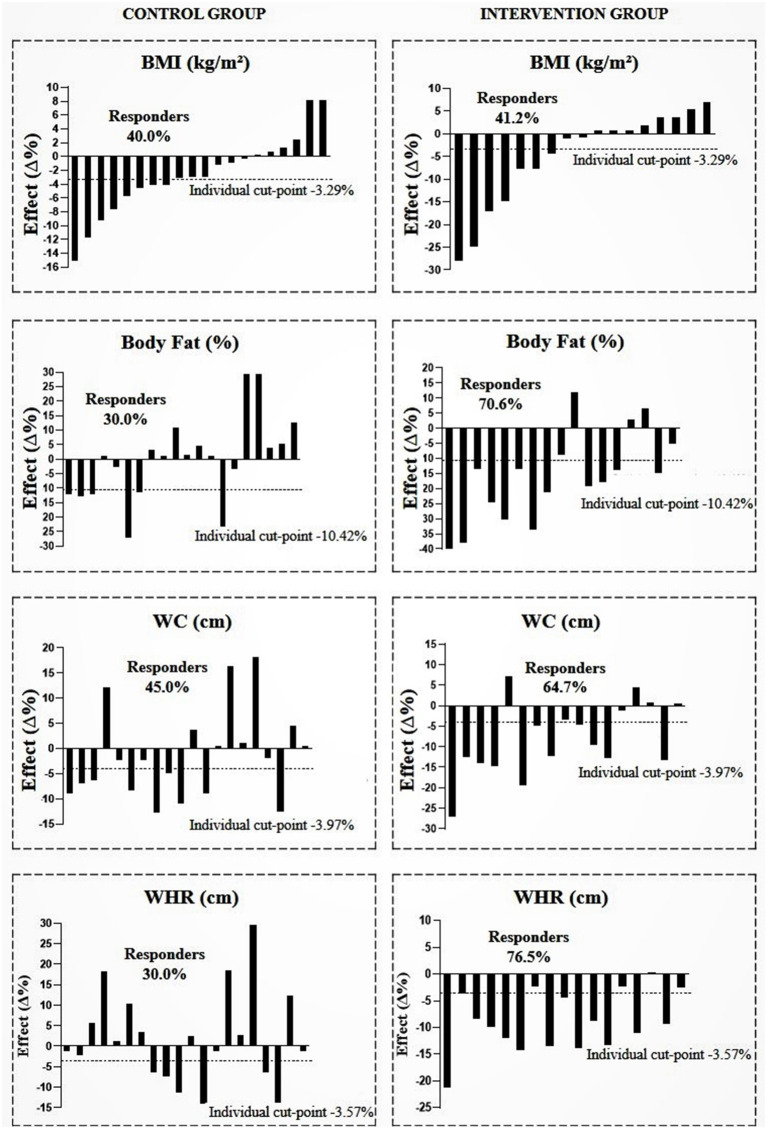
Prevalence of responders in the control and intervention group after multicomponent intervention for body mass index (BMI), body fat, waist circumference (WC), and waist/hip ratio (WHR). CG, *n* = 20; IG, *n* = 17. The analyses were adjusted for sex, age, and sexual maturation.

**Figure 3:**
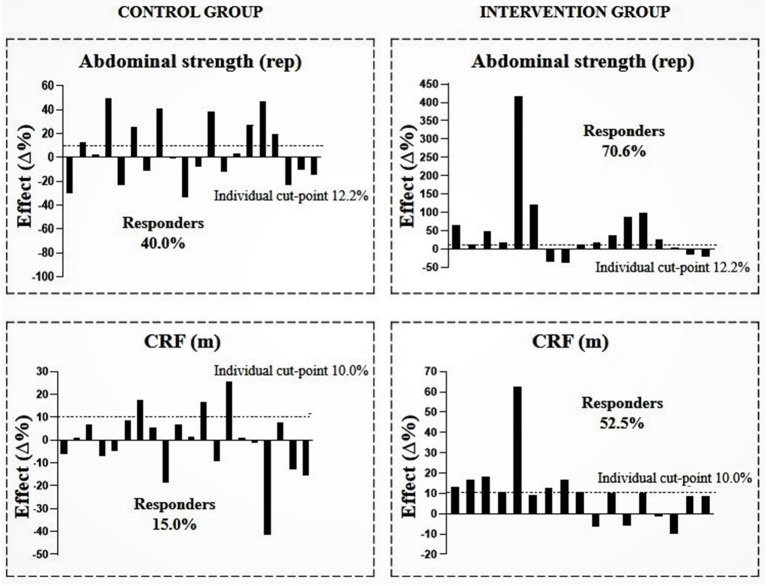
Prevalence of responders in the intervention and control group after multicomponent intervention for abdominal strength and cardiorespiratory fitness (CRF). CG, *n* = 20; IG, *n* = 17. The analyses were adjusted for sex, age, and sexual maturation.

**Figure 4:**
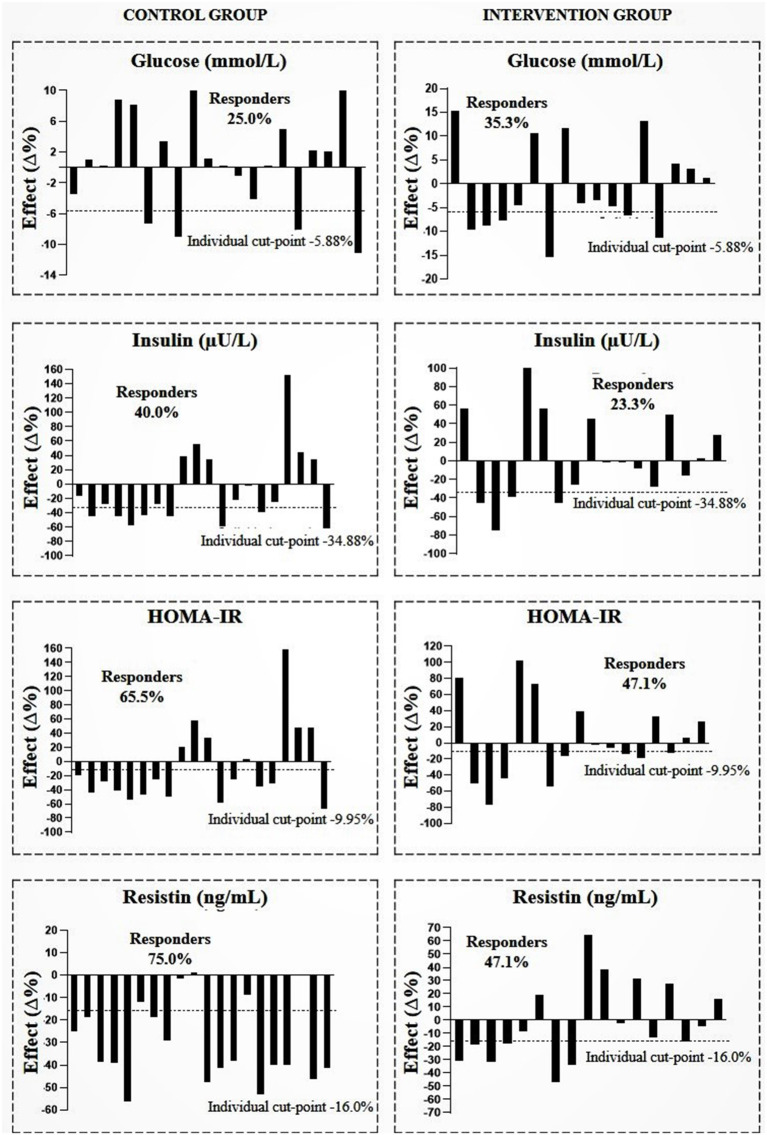
Prevalence of responders in the intervention and control group after multicomponent intervention for glucose, insulin, homeostasis model assessment of insulin resistance (HOMA-IR), and resistin. CG, *n* = 20; IG, *n* = 17. The analyses were adjusted for sex, age, and sexual maturation.

Figures 2–4 show the prevalence of individuals who responded positively to the multicomponent intervention process according to the pre-established cutoff points for each variable, presented in the method of the present study. The CG graphs are shown on the left and the IG graphs are shown on the right; both groups present those evaluated in equal positions for all graphs. Regarding the prevalence of respondents between the control and intervention groups, significant differences were found in body fat (CG = 30%; IG = 70.6%; PR = 1.396; *p* < 0.001), WHR (CG = 30%; IG = 76.5%; PR = 1.730 *p* < 0.001), and CRF (CG = 15%; IG = 52.5%; PR = 1.580; *p* ≤ 0.001); the other variables did not show differences in the prevalence of responders (Table 3).

**Table 3:** Differences in the prevalence of responders between IG and CG.

	**Responders**		
	**PR**	**95% CI**	***P***
**CG** **×** **IG**			
BMI (kg/m^2^)	0.911	0.668–1.241	0.554
Body fat (%)	1.396	1.158–1.684	<0.001
WC (cm)	1.173	0.869–1.584	0.296
WHR (cm)	1.730	1.321–2.265	<0.001
CRF (m)	1.580	1.213–2.058	<0.001
Abdominal strength (rep)	1.209	0.892–1.638	0.221
Glucose (mmol/L)	1.189	0.902–1.566	0.219
Insulin (μU/L)	0.773	0.567–1.054	0.103
HOMA-IR	0.787	0.566–1.094	0.154
Resistin (ng/ml)	0.806	0.590–1.103	0.175

*BMI, body mass index; WC, waist circumference; WHR, waist-to-height ratio; CRF, cardiorespiratory fitness; HOMA-IR, homeostasis model assessment of insulin resistance. CG, control group; IG, intervention group; Poisson regression; PR, prevalence ratio; p: significance level <0.05; adjusted for sex, age, and sexual maturation*.

## Discussion

The present study evaluated the effects of a 6-month multicomponent intervention program based on aerobic and resistance physical exercises (sports, functional circuit, recreational, and water activities), with nutritional recommendations and psychological support on body composition, aerobic, and muscular physical fitness and insulin biomarkers in overweight and obese adolescents. There was a significant effect on the prevalence of responders for body fat (70.6%), WHR (76.5%), and CRF (52.5%) of the adolescents participating in the intervention concerning CG.

The search for evidence on the effects of physical exercise on overweight and obesity in children and adolescents is broad, given that the reduction in body measures is related to the cardiometabolic risks (Rajjo et al., 2016). An intervention study of 28 weeks of high-intensity interval training (HIIT) during physical education classes with overweight and obese adolescents conducted by Delgado-Floody et al. (2018) showed reduction in BMI, WC, WHR, and body fat after the intervention. In the present study, there was a significant prevalence of responders for body fat (70.6%) and WHR (76.5%) in the IG who performed exercises considered to be of moderate intensity. Thus, it is expected that after an intervention program with moderate to high physical exercises and with different modalities, it is possible to decrease the parameters of body composition. It should be noted that these are indicators of metabolic risk and that inadequate values directly affect the health of adolescents, which can have long-term consequences (Bridger, 2009; Jankowski et al., 2015).

In our study, the IG also showed a significant prevalence of responders for CRF, in which 52.9% of participants presented an improvement after the intervention. An intervention study carried out with obese adolescents showed significant improvements in aerobic indexes and cardiovascular efficiency within only 12 weeks of exercise interspersed with sports, circuits, and swimming three times a week (Klijn et al., 2007). In the study by Brand et al. (2019), overweight and obese children had no effect on CRF after a multicomponent intervention program with intense exercise sessions twice a week for 12 weeks. Indeed, CRF is considered a predictor of mortality and a physiological, psychosocial, and cognitive indicator in children and adolescents (Lang et al., 2018). Therefore, Sigal et al. (2014) consider that at least three sessions of aerobic or combined physical exercise of moderate to high intensity per week are sufficient to obtain an improvement in CRF of overweight or obese adolescents. Improving aerobic and anaerobic fitness in children and adolescents is indeed encouraging, given the low levels of physical fitness in this population today. The effects of interventions that aim to improve this parameter may be related to the content and structure of the applied exercise sessions. Developing sessions focused on motor skills, strength training, and physical conditioning appropriate to the profile of the participants can be a positive strategy, since pleasurable activities promote feelings of motivation that contribute to a better commitment (Bonney et al., 2019).

The present study had no significant effects on the variables glucose, insulin, and HOMA-IR. The same was observed in the study by Sigal et al. (2014), which demonstrated that there were no effects on changes in lipid and glucose levels after a combined 4-week exercise program. The lack of effect on these markers could be due to values at baseline that were in the normal range and reduces the capacity of improvement; this occurred in our study with the variable HOMA-IR, and most of the participants were classified as normal for that parameter in the baseline. In a recent study that included sports, psychotherapy, and nutritional counseling, HOMA-IR levels in obese children and adolescents decreased, demonstrating that this was a sensitive parameter for a short-term intervention (5 months) (Mayerhofer et al., 2020). According to Zhai et al. (2015) and Wagner et al. (2013), insulin resistance may be influenced by the stage of maturation, and excess weight is already known as a modulator of sexual maturation, causing the onset of puberty at an early stage. HOMA-IR levels vary physiologically according to age and are higher when there is a diagnosis of obesity; the peak can be reached between 13 and 15 years of age and after, and return to normal levels at the end of the maturation process (Shashaj et al., 2016). Therefore, the cutoff values for HOMA-IR may be higher in individuals during the puberty process than in individuals who have not yet entered the maturation process (Kurtoglu et al., 2010).

In addition to having no effect, when observing the prevalence of responders in the variables insulin and HOMA-IR, response in the CG was greater compared to the GI in the present study. Mayerhofer et al. (2020) studied the correlation of BMI and body fat with changes in HOMA-IR and did not find any association of these parameters, suggesting that the decrease in HOMA-IR levels during the physical exercise program and nutritional guidance is independent of changes in corporal composition. Therefore, there is a possibility that the short-term effects of exercise may increase the uptake of glucose in muscle tissue, causing a decrease in insulin secretion and lower HOMA-IR levels. However, some studies have found associations between changes in BMI with HOMA-IR after long-term (over 6 months) intervention programs (Kalavainen et al., 2012; Santos et al., 2015). On the other hand, there is evidence that liver fat may be an independent determinant for HOMA-IR in adolescents, and not visceral or total fat (Linder et al., 2014). Therefore, it could be assumed that there is a mechanism different from HOMA-IR levels for overweight and obese adolescents.

Resistin was a marker that, despite not showing significant differences between groups, unexpectedly showed higher prevalence of responders for CG adolescents (75.0%). Gerber et al. (2005) concluded that resistin level in children and adolescents shows a correlation with pubertal stage and age. In thin boys, this correlation is positive, while in girls, this correlation is significant and positive only when obese girls are included in the analysis. These results are confirmed by the correlation of resistin with testosterone in thin and obese boys and with estradiol in obese girls. An increase in resistin levels during pubertal maturation is also supported by a progressive multiple regression model, including age, Tanner stage, estradiol, testosterone, WC, WHR, BMI, weight, and height. Tanner's stage was the only significant independent predictor for resistin, explaining 11% (*p* < 0.001) of its variance. Considering that the correlations between resistin and BMI were of low significance for the obese group and absent in the lean study group, it is suggested that the parameters of pubertal maturation are the strongest variation of the resistin and that only states of morbid obesity are associated with high levels of resistin (Savage et al., 2001). These findings may justify what happened in our study, in which adolescents are at different stages of maturation and within a wide age range, explaining the lack of effect and the unexpected response in insulin biomarkers.

In this sense, Nascimento et al. (2016) did not find changes in resistin after an intervention program in school physical education, but found a positive correlation between resistin, triglycerides, and TNF-alpha at baseline, and that higher concentration was associated with increased other inflammatory markers, including IL-6. Therefore, according to Bokarewa et al. (2005) and Lehrke et al. (2004), it is suggested that resistin is produced in response to other inflammatory stimuli and induces the synthesis of other pro-inflammatory cytokines. With respect to the relationship between insulin resistance and cytokines, Rubin et al. (2008) sought to find associations between vigorous physical activity and HOMA-IR with some inflammatory markers. Regarding resistin, vigorous physical activity interacted opposite to HOMA-IR, indicating that the increase in resistin induces an increase in HOMA-IR in individuals with high levels of vigorous physical activity. No weight status and general adiposity, as indicated by BMI, puberty, or ethnicity, explained this association. Therefore, it is clear that the evidence on resistin is still inconsistent. Further clinical studies aimed at investigating the expression and manifestation of this protein are needed to determine its mechanism within the inflammatory process of obesity.

The effects of excess weight, more specifically obesity, indicate a higher prevalence of cardiometabolic risk factors. However, studies showed a good prognosis for overweight or slightly obese individuals, calling this the obesity paradox. This paradox may be related to unmeasured confounding factors, such as unintentional weight loss in individuals who do not participate in exercise programs or obesity treatment (Lavie et al., 2013, 2014, 2015). Also, genetic characteristics may be involved in this paradox, meaning that individuals closer to having an adequate body composition may present a less favorable clinical profile concerning blood pressure, lipid, glycemic, and inflammatory parameters. That is, the etiology and genetic disposition may be associated with the unfavorable clinical condition (Lavie et al., 2016).

Our multicomponent intervention program with varied exercises (sports, functional circuit, recreational, and water activities) and nutritional and psychological guidance was effective for the body composition parameters (body fat and WHR) and for the CRF, therefore emphasizing the importance of programs with multicomponent approaches (physical exercise and nutritional and psychological orientation) with efforts aimed at the profile of individuals and with the collaboration of a community body that aims at an integrated service of a multidisciplinary nature.

## Limitations

The present study has some limitations, such as the limited sample size to obtain a medium effect, making it impossible to detect small effects, and not having assessed the levels of physical activity of participants in the control and intervention groups to determine whether the effects presented were directly caused by the exercise program. It was also not possible to control eating behavior, as the program only offered guidance on eating habits. We did not perform correlation analysis to find out how the variables are related. However, the study has several strengths, since most studies to date assess the relationship between adiposity and biochemical and physical fitness markers in an observational way. In addition, the study not only assessed the effect of the intervention program but also considered the response to adolescents' inter-individual variability. It is also relevant because it is one of the few studies that evaluated resistin as a biomarker of insulin in the pediatric population.

## Conclusion

The 6-month multicomponent intervention program with varied exercises (sports, functional circuit, recreational, and water activities) and nutritional and psychological guidance improved certain parameters of body composition and the CRF of overweight and obese adolescents but did not improve insulin biomarkers. The present results suggest that other indicators, such as age and maturation stage, may play a more important role regarding the effects of the intervention on insulin biomarkers in overweight and obese adolescents, highlighting mainly that resistin is still an inflammatory marker with an inconsistent mechanism in this relationship. Therefore, this intervention model can be considered and adapted for schools and community establishments as a way to improve the indicators of body composition and CRF in overweight and obese adolescents.

## Data Availability Statement

The raw data supporting the conclusions of this article will be made available by the authors, without undue reservation.

## Ethics Statement

The studies involving human participants were reviewed and approved by all procedures were approved by the Scientific Council of the Research Unit that leads the project under number CAAE: 54985316.0.0000.5343 and opinion number: 1.498.338 and are registered at Clinical Trials under Protocol ID: 54985316.0.0000.5343. Written informed consent to participate in this study was provided by the participants' legal guardian/next of kin.

## Author Contributions

LBorb, CP, AG, JB-S, and CB were responsible for the conception and design, data acquisition, data analysis and interpretation, writing of the initial article, and critical review of important intellectual content. LBorf and JD were responsible for the conception and design, data acquisition, and review of all the minutes of the article. All authors read and approved the final article.

## Conflict of Interest

The authors declare that the research was conducted in the absence of any commercial or financial relationships that could be construed as a potential conflict of interest.
