# Supplementary material for: A Multicomponent Intervention Program With Overweight and Obese Adolescents Improves Body Composition and Cardiorespiratory Fitness, but Not Insulin Biomarkers
Source: Front Sports Act Living. 2021 Feb 22;3:621055. doi: 10.3389/fspor.2021.621055 (PMC7937702; doi:10.3389/fspor.2021.621055)
Supplement: Supplementary file 1 [file Data_Sheet_1.PDF]

Frame 1. Exemplification of the mesocycle and macrocycle

**1º Period**		
	**Objectives**	**Methods**
**Mondays**	- Perform a 30-minute walk, keeping the heart rate in the indicated area (50% to 60% of the maximum HR);<br>- Perform sports activities.	- Heating;<br>- Aerobic walk;<br>- Sports activities;<br>- Stretching.
**Wednesdays**	- Adaptation to the aquatic environment;<br>- Perform anaerobic exercises;<br>- Work on controlled exhalation and breathing rhythm;<br>- Perform initiation to swimming.	- Heating;<br>- Pedagogical processes (ventral and dorsal slide; alternate beating of the ventral and dorsal lower limbs; propulsion of the upper limbs; immersions with control and breathing rhythm; transverse and longitudinal axis rotations);<br>- Stretching.
**Fridays**	- Maintain heart rate in the indicated area (50% to 60% of maximum HR);<br>- Development muscular endurance and strength;<br>- Increase flexibility;<br>- Improvement of lung capacity and respiratory function.	- Breathing exercises;<br>- Heating;<br>- Circuit of aerobic and resistance exercises;<br>- Aerobic walk;<br>- Stretching.
**2º Period**		
	**Objectives**	**Methods**
**Mondays**	- Perform a 30-minute walk, keeping the heart rate in the indicated area (50% to 60% of the maximum HR);<br>- Perform sports activities.	- Heating;<br>- Aerobic walk;<br>- Sports activities;<br>- Stretching.
**Wednesdays**	- Perform anaerobic exercises;<br>- Work on controlled exhalation and breathing rhythm;<br>- Perform the fundamentals of swimming;	- Heating and stretching;<br>- Pedagogical processes (ventral and dorsal slide; alternate beating of the ventral and dorsal lower limbs; propulsion of the upper limbs; immersions with control and breathing rhythm; transverse and longitudinal axis rotations);<br>- Stretching.
**Fridays**	- Maintain heart rate in the indicated area (50% to 60% of maximum HR);<br>- Development muscular endurance and strength;<br>- Increase flexibility;<br>- Improvement of lung capacity and respiratory function.	- Breathing exercises;<br>- Heating;<br>- Circuit of aerobic and resistance exercises;<br>- Aerobic walk;<br>- Stretching.
**3º Period**		
	**Objectives**	**Methods**
**Mondays**	- Perform a 30-minute walk, keeping the heart rate in the indicated area (50% to 60% of the maximum HR);<br>- Perform sports activities.	- Heating;<br>- Aerobic walk;<br>- Sports activities;<br>- Stretching.
**Wednesdays**	- Perform anaerobic exercises;<br>- Work on controlled exhalation and breathing rhythm;<br>- Deepen the fundamentals of swimming;	- Aquecimento e alongamento;<br>- Pedagogical processes (ventral and dorsal slide; alternate beating of the ventral and dorsal lower limbs; propulsion of the upper limbs; immersions with control and breathing rhythm; transverse and longitudinal axis rotations);<br>- Stretching.

**Friday**	- Maintain heart rate in the indicated area (50% to 60% of maximum HR);<br>- Development muscular endurance and strength;<br>- Increase flexibility;<br>- Improvement of lung capacity and respiratory function.	- Breathing exercises;<br>- Heating;<br>- Circuit of aerobic and resistance exercises;<br>- Aerobic walk;<br>- Stretching.

Frame 2. Distribution of physical exercise session

**Periods**	**Mondays**	**Wednesdays**	**Fridays**
**Session time**	10 min heating<br>30 min walk<br>15 min sports activities<br>5 min stretches	10 min heating<br>45 min water activities<br>5 min stretches	15 min breathing exercises<br>10 min heating<br>40 min circuit (resisted and aerobic)<br>45 min walk<br>10 min stretches
**Total**	1 hour	1 hour	2 hours

*Min: minutes.
